# CCK Response Deficiency in Synphilin-1 Transgenic Mice

**DOI:** 10.1371/journal.pone.0142314

**Published:** 2015-11-16

**Authors:** Wanli W. Smith, Megan Smith, Dejun Yang, Pique P. Choi, Alexander Moghadam, Tianxia Li, Timothy H. Moran

**Affiliations:** 1 Department of Pharmaceutical Sciences, University of Maryland School of Pharmacy, Baltimore, Maryland, United States of America; 2 Department of Psychiatry, Johns Hopkins University School of Medicine, Baltimore, Maryland, United States of America; National Institute of Agronomic Research, FRANCE

## Abstract

Previously, we have identified a novel role for the cytoplasmic protein, synphilin-1(SP1), in the controls of food intake and body weight in both mice and Drosophila. Ubiquitous overexpression of human SP1 in brain neurons in transgenic mice results in hyperphagia expressed as an increase in meal size. However, the mechanisms underlying this action of SP1 remain to be determined. Here we investigate a potential role for altered gut feedback signaling in the effects of SP1 on food intake. We examined responses to peripheral administration of cholecytokinin (CCK), amylin, and the glucagon like peptide-1 (GLP-1) receptor agonist, exendin-4. Intraperitoneal administration of CCK at doses ranging from 1–10 nmol/kg significantly reduced glucose intake in wild type (WT) mice, but failed to affect intake in SP1 transgenic mice. Moreover, there was a significant attenuation of CCK-induced c-Fos expression in the dorsal vagal complex in SP1 transgenic mice. In contrast, WT and SP1 transgenic mice were similarly responsive to both amylin and exendin-4 treatment. These studies demonstrate that SP1 results in a CCK response deficiency that may contribute to the increased meal size and overall hyperphagia in synphillin-1 transgenic mice.

## Introduction

Obesity is the most common metabolic disease and its worldwide prevalence makes obesity a major health problem. The pathogenesis of obesity remains incompletely understood. We have identified a novel role for SP1 in the control of food intake and body weight in transgenic mice and flies [1–4]. Ubiquitous overexpression of SP1 in brain neurons results in hyperphagia and obesity in both mice and Drosophila. SP1, a 919 amino acid protein, was first identified as an alpha-synuclein interacting protein by yeast two hybrid analysis [5]. SP1 is predominantly expressed in the cytosol in many tissue, with enriched expression in neurons in the brain [5]. The function of SP1 is not fully understood. Previously, we and others have demonstrated that SP1 associates with alpha-synuclein promoting the formation of intracellular inclusions, and plays a protective role against neuronal toxicity in Parkinson’s disease cell and mouse models [5–10]. Our recent studies have shown that SP1 binds ATP and GTP, and increases cellular energy levels in cultured cells [3]. Moreover, SP1 is expressed at a high level in both neuronal cell bodies and nerve terminals in the hypothalamus [1], which is a major neural regulator of energy balance. Thus, investigating the actions of SP1 may provide further novel insight into the understanding of the molecular mechanisms of energy homeostasis.

Characterization of human SP1 transgenic mice has demonstrated that neuronal expression of SP1 increased body weight gain and fat deposition secondary to increased food intake [1]. Meal pattern studies have shown that the increased food intake was expressed as a significant increase in average meal size without a change in meal number [4]. How SP1 alters meal size is not clear. The controls of food intake behavior are complex. A major control of meal size is within meal gut feedback signaling arising from the presence of ingested nutrients in the gastrointestinal tract. Multiple gut peptides have been identified to play roles in the control of meal size. Previous studies have revealed that deficits in post-ingestive inhibitory feedback can result in an increase in meal size [11]. Satiety gut peptides such as cholecytokinin (CCK), amylin, and glucagon like peptide-1 (GLP-1), among others, have been shown to reduce meal size following their exogenous administration [12–14], and the endogenous release of each has been demonstrated to play a role in meal feedback signaling. Administration of CCK, amylin, and GLP-1 antagonists result in increases in meal size [15–17]. Thus, in this study, we proposed to test the hypothesis that neuronal overexpression of SP1 in mice alters brain ability to respond to inhibitory satiety gut peptides (CCK, amylin, and GLP-1) resulting in increased meal size.

To assess the changes in responses to gut feedback signaling, WT and human SP1 transgenic mice were given CCK, amylin, and GLP-1 peripherally. The feeding behavior and brain neuronal response were compared to determine whether SP1 mice have a deficit in satiety signaling that may be contributing to an alteration in the ability to limit the size of meals.

## Materials and Methods

### Animals

The SP1 mice were generated as described previously [1,10], in which human SP1 was predominantly expressed in neurons under the mouse prion protein promoter [10]. The experimental SP1 mice were generated from successive backcrossing with the C57BL6 strain.

The WT and SP1 mice were identified by PCR-genotyping at 3 weeks of age as described previously [1,10]. SP1 mice at 6 weeks of age were classified as “pre-obese,” and those at 5 months of age were classified as “obese.” All animal experiments were approved by the Johns Hopkins University Institutional Animal Care and Use Committee. To further verify SP1 expression in SP1 mouse brain, the brain homogenates from WT and SP1 mice were subjected to western blot analysis using anti-SP1 antibodies as described previously [1,10].

### Gut peptide administration

Amylin was from Phoenix Pharmaceuticals (Burlingame, CA, USA). CCK (native tyrosine-sulfated form which can bind to both CCK A and B receptors) and Exendin-4 were from Bachem (King of Prussia, PA, USA). Mice were maintained in our meal pattern monitoring cages. Mice were placed in a schedule of 2 hour food deprivation immediately prior to lights out. On test days mice were injected intraperitoneally (ip.)with various doses of CCK (0, 1, 3.2, 5 and 10 nmol/kg) and the GLP-1 agonist, exendin-4 (0, 1 and 3.2 nmol/kg) as described [18–20]. Amylin (0, 7.5 and 15 μg/kg) was injected subcutaneously (s.c.) as described previously [21] given that amylin s.c. administration results in both a more steady and effective food intake response compared with i.p. administration. A liquid diet-glucose (25%) was given after the injection. The glucose intake was measured 30 min following peptide administration. Two days of vehicle administration occurred between doses. Doses were administered in random order. Responses to gut peptide administration were assessed at pre, and obese stages at 6 weeks and 5 months using different cohorts of mice for each peptide and developmental time point. There were nine mice for each experimental group.

### Immunohistochemistry analysis and c-Fos expression assay

Pre-obese and obese SP1 mice and control WT mice were used for CCK-linked c-Fos expression experiments. Brains were perfused with 4% paraformaldehyde. Frozen brain sections through the area postrema (AP) and rostrocaudal extent of the NTS were subjected to immunohistochemistry staining using anti-c-Fos antibodies as described previously [22]. Briefly, sections were probed with anti- c-Fos antibodies as a primary antibody (rabbit polyclonal, Oncogene) followed by standard immunoperoxidase methods using ABC kit (Vector Laboratories). The Ni-3′3′-diaminobenzidine (DAB, Vector Laboratories) was used as a detection agent. The matched sections were incubated without primary antibodies as negative controls. c-Fos immunoreactivity was quantified using the IP Laboratory Imaging System (Scanalytics, Vienna, VA) image analysis software. The c-Fos-positive cells were counted by an investigator who was blind to the experimental conditions. The number of c-Fos-positive cells were counted across the rostral-caudal levels (i.e., caudal, medial, intermediate, and rostral) and in the coronal bilateral sections from four rostrocaudal levels of the NTS and AP in each mouse. The coordinates for the above areas are as follows with respect to the interaural line [23]: the caudal NTS area (−5.6 mm), medial NTS area (−5.06 mm), intermediate NTS area (−4.3 mm), and rostral NTS area (−3.8 mm). This analysis provided a view of c-Fos activation in the rostral-caudal NTS area.

### Data analysis

Quantitative data from western blot, food intake, body weight, and c-Fos cells were expressed as arithmetic means ± SEM. Comparisons of effects of gut peptides among two genotype groups in food intake and c-Fos cell numbers were analyzed using two-way analysis of variance (ANOVA) with Sigmastart 3.1 statistical software (Aspire Software International, VA). Post-ANOVA analyses of group differences were performed with the Tukey test. A p value <0.05 will be considered significant.

## Results

### Neural SP1 overexpression did not alter the response to amylin

**Fig 1:**
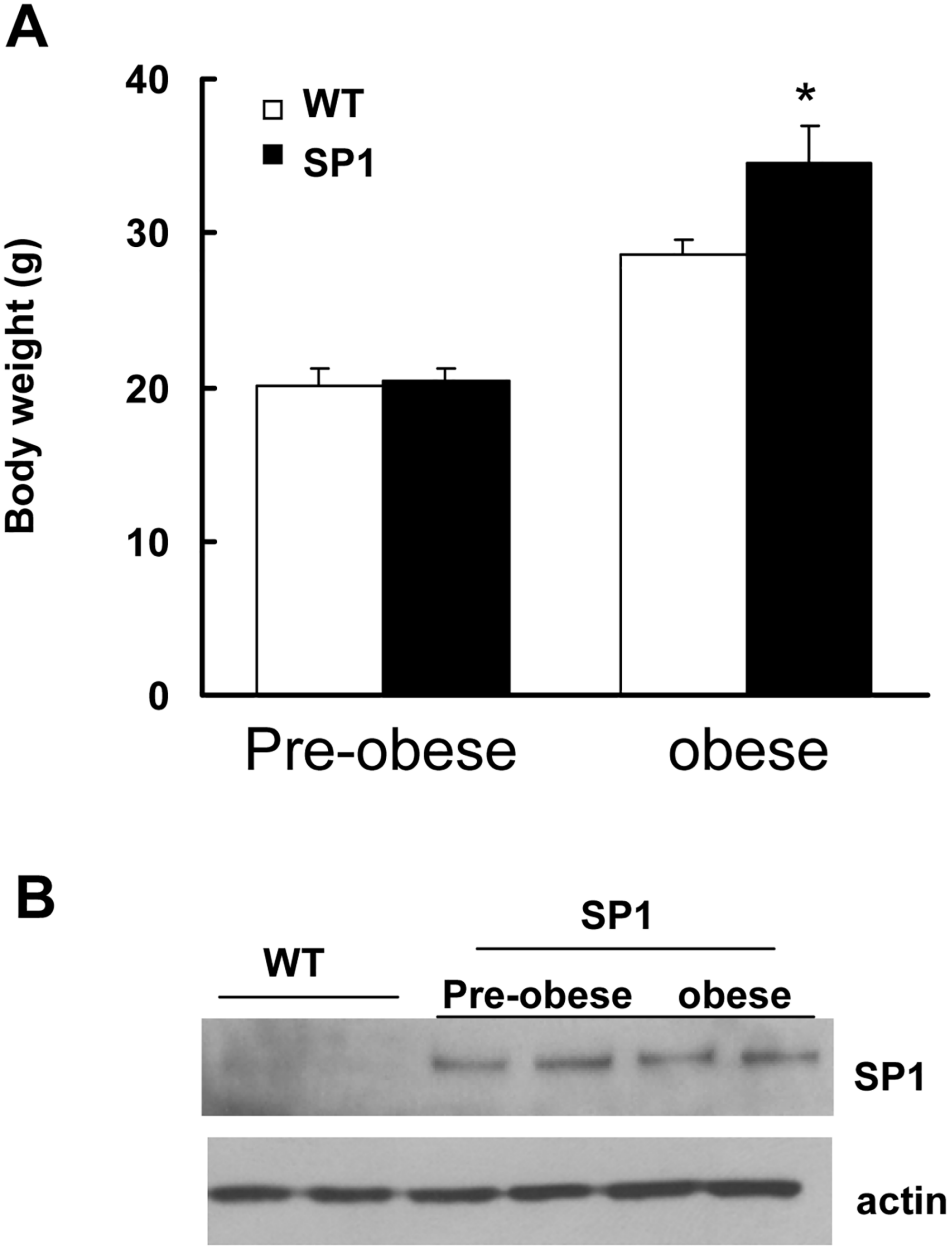
SP1 transgenic mice at pre-obese and obese stages. A. body weight. *P*<0.05 by ANOVA, vs WT mice. B. Western blots showing SP1 expression in brains in SP1 mice.

**Fig 2:**
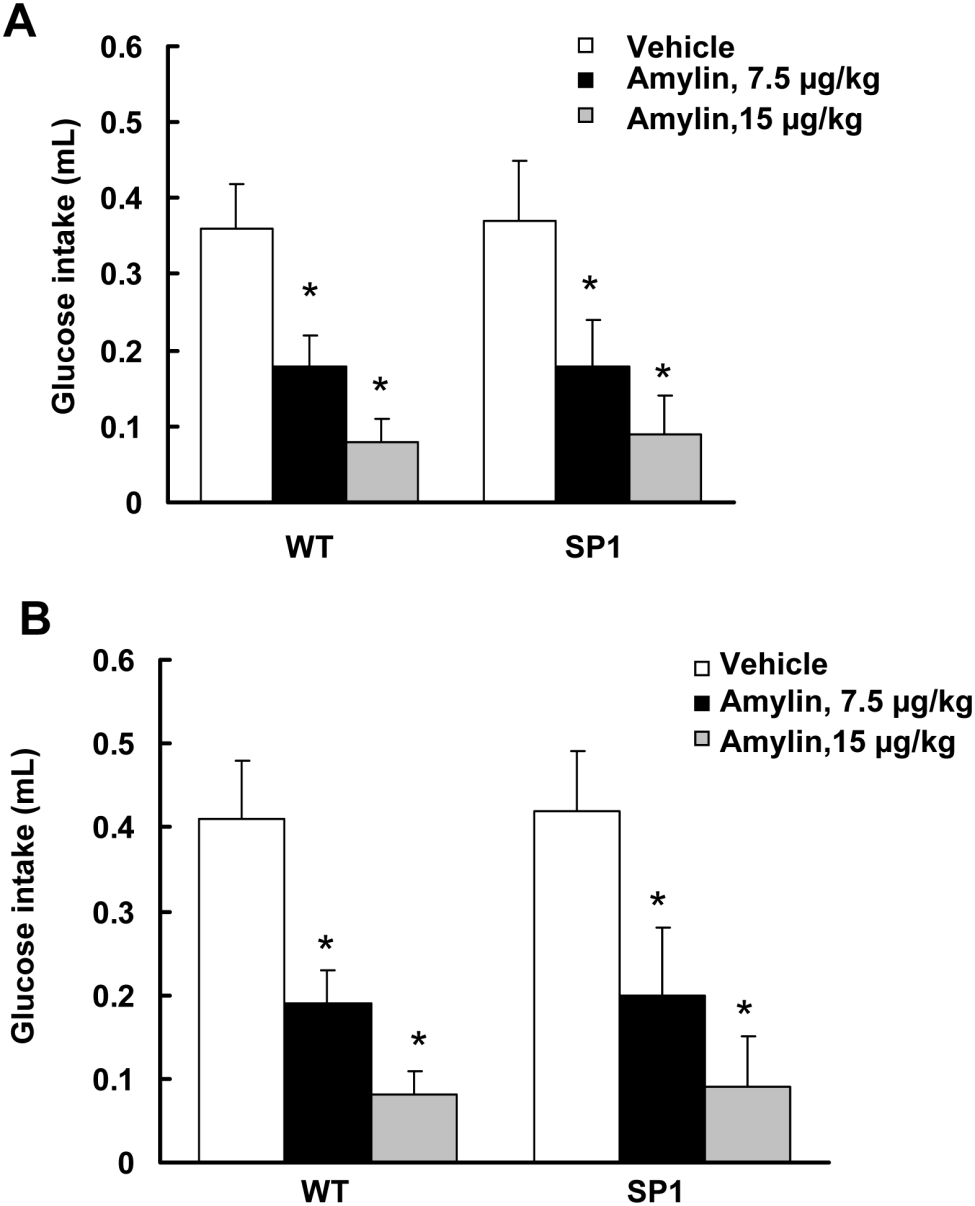
SP1 did not alter anorectic effects of amylin. A. Effect of amylin on pre-obese SP1 mice. B. Effects of amylin on obese SP1 mice. *P<0.05 by two-way ANOVA, vs untreated WT or untreated SP1 mice.

SP1 mice at 6 weeks of age did not display a body weight difference compared with WT mice (Fig 1A). Consistent with previous findings, SP1 mice at 5 month of age showed significant body weight gain compared with WT (Fig 1A). Both pre-obese and obese SP1 mice overexpressed human SP1 protein at steady levels in brains (Fig 1B). Amylin is an islet amyloid polypeptide released by the pancreas comprised of 37 amino acids. Amylin decreases food intake by reducing meal size and slowing gastric emptying [12]. To assess whether overexpression of SP1 alters the response to amylin, a glucose intake assay was performed using WT and SP1 mice at 6 weeks (pre-obese) and 5 months of age (obese stage). There was no difference in 30-min glucose intake between SP1 mice and WT mice at both pre-obese and obese stage (Fig 2). Amylin reduced 30 min-glucose intake in both WT and SP1 mice in a dose-dependent manner (Fig 2A and 2B). There was no difference in the response to amylin between the genotypes of mice.

### Neural SP1 overexpression did not alter the response to exendin-4

**Fig 3:**
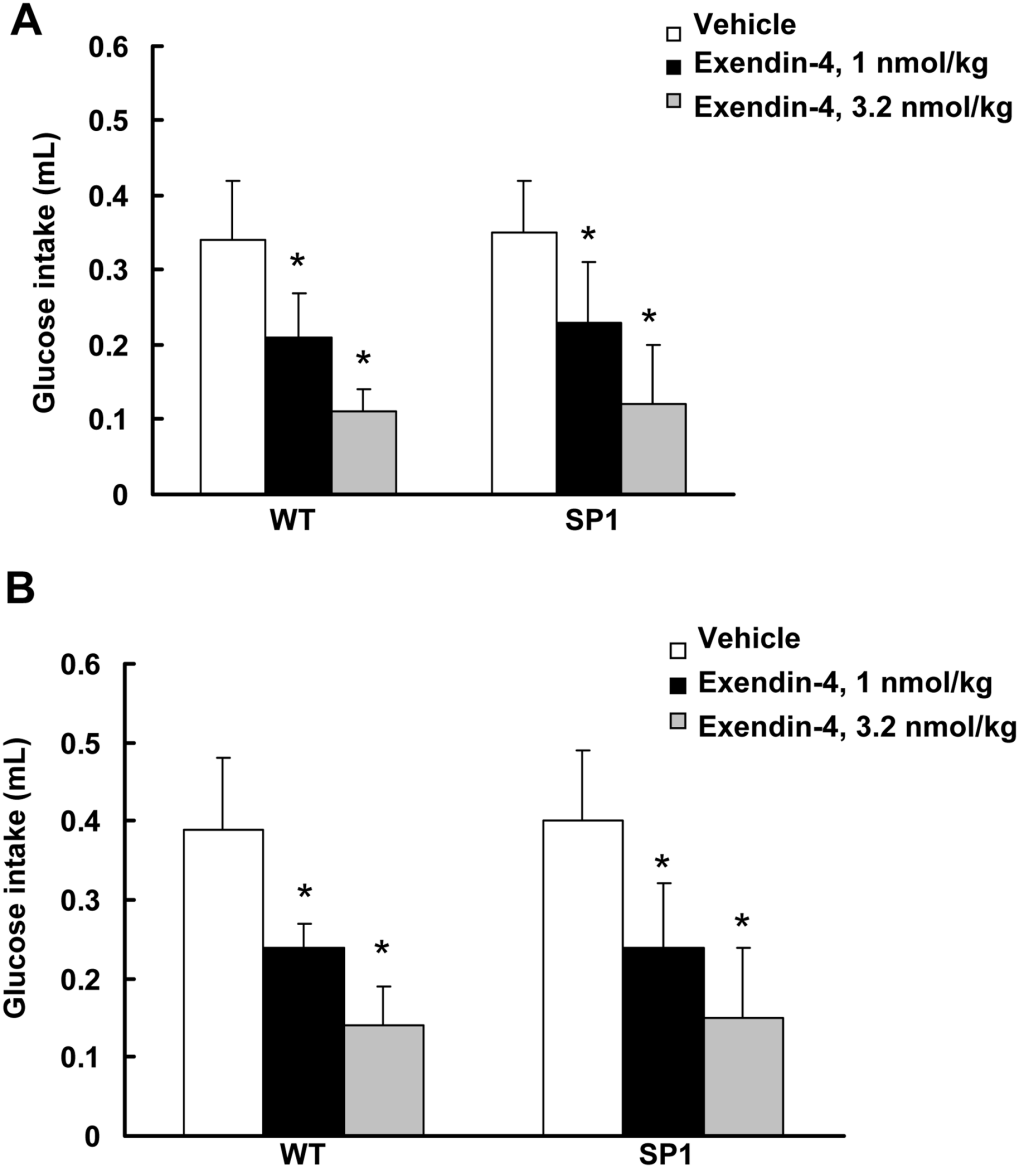
SP1 did not alter anorectic effects of exendin-4. A. Effect of exendin-4 on pre-obese SP1 mice. B. Effects of exendin-4 on obese SP1 mice. *P<0.05 by two-way ANOVA, vs untreated WT or untreated SP1 mice.

Glucagon-like peptide-1 (GLP-1) is secreted from the intestine during food ingestion. Peripheral treatment with GLP-1 decreases food intake in both rodents and humans [13]. GLP-1 is rapidly degraded in circulation, with a half-life of about 1 minute. Thus, exendin-4 (1–39), a GLP-1 receptor agonist with a relatively longer half-life [24], was used to assess whether SP1 alters the response to GLP-1 receptor activation. Administration of exendin-4 significantly reduced 30 min-glucose intake in both WT and SP1 mice (Fig 3). Compared with saline vehicle control group, both pre-obese and obese SP1 mice responded well to exendin-4 (1–39) treatment with reduction of glucose intake. However, there were no response differences in glucose uptake between the WT and SP1 mice after exendin-4 administration (Fig 3).

### Neural SP1 overexpression caused a deficit in responding to CCK

**Fig 4:**
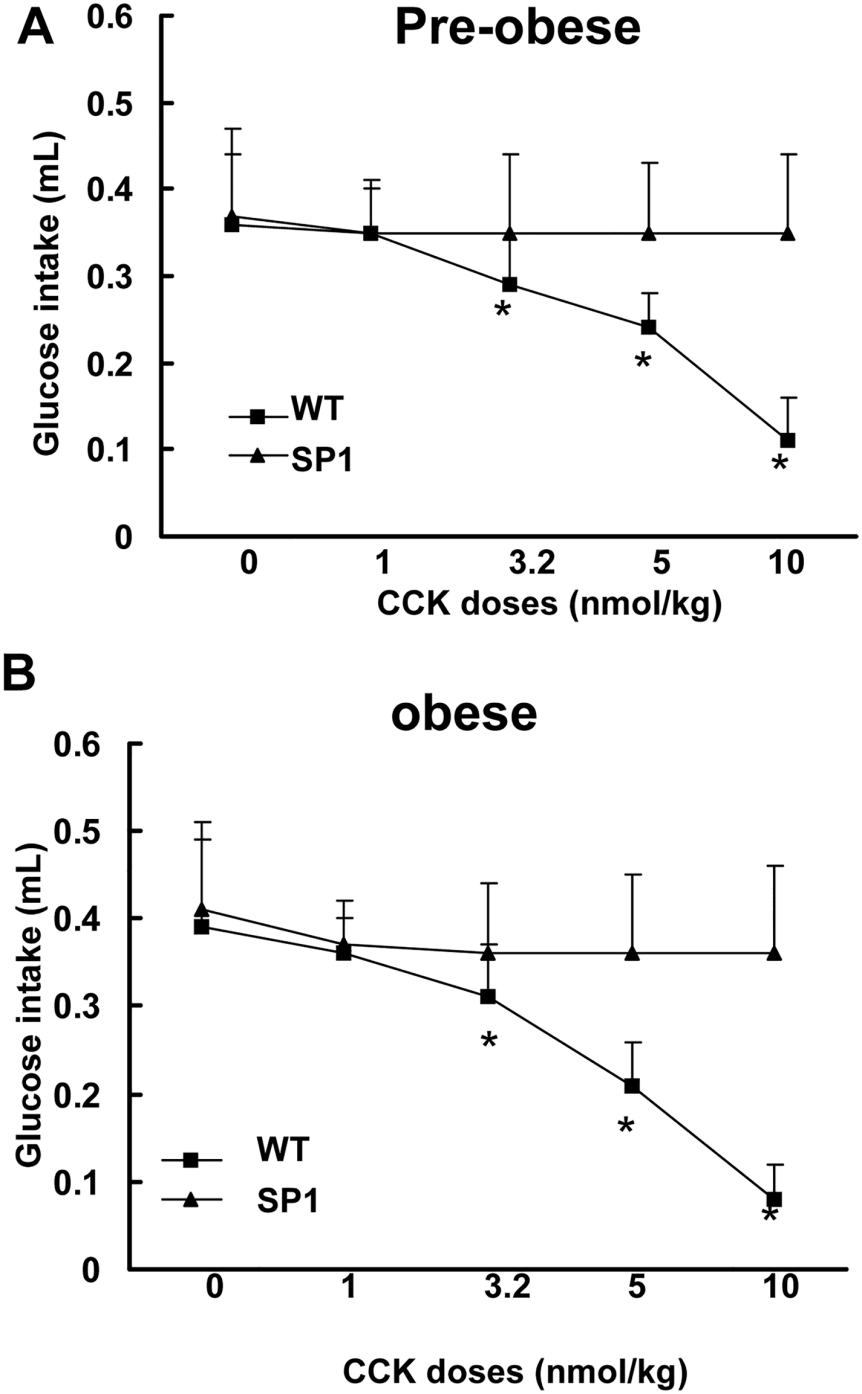
SP1 altered CCK-induced anorectic effects in SP1 mice. A. Effect of CCK on pre-obese SP1 mice. B. Effects of CCK on obese SP1 mice. * *P*<0.05 by two-way ANOVA, vs untreated WT or untreated SP1 mice.

**C**holecystokinin (CCK) is an anorectic gastrointestinal hormone released from the proximal small intestine in response to nutrient consumption [14]. CCK satiation is mediated through the activation of CCK 1 receptors on vagal afferent fibers [25]. To assess whether neuronal overexpression of SP1 alters the response to CCK, both WT and SP1 mice were injected (*i*.*p*.) with CCK at doses of 1–10 nmol/kg. Treatment with CCK in WT mice significantly reduced 30-min glucose intake in a dose-dependent fashion compared with untreated control mice (Fig 4). In contrast, there was a lack of response to CCK treatment in SP1 mice at both pre-obese and obese stages in glucose intake assays.

### SP1 alters CCK-induced c-Fos expression in dorsal vagal complex (DVC)

**Fig 5:**
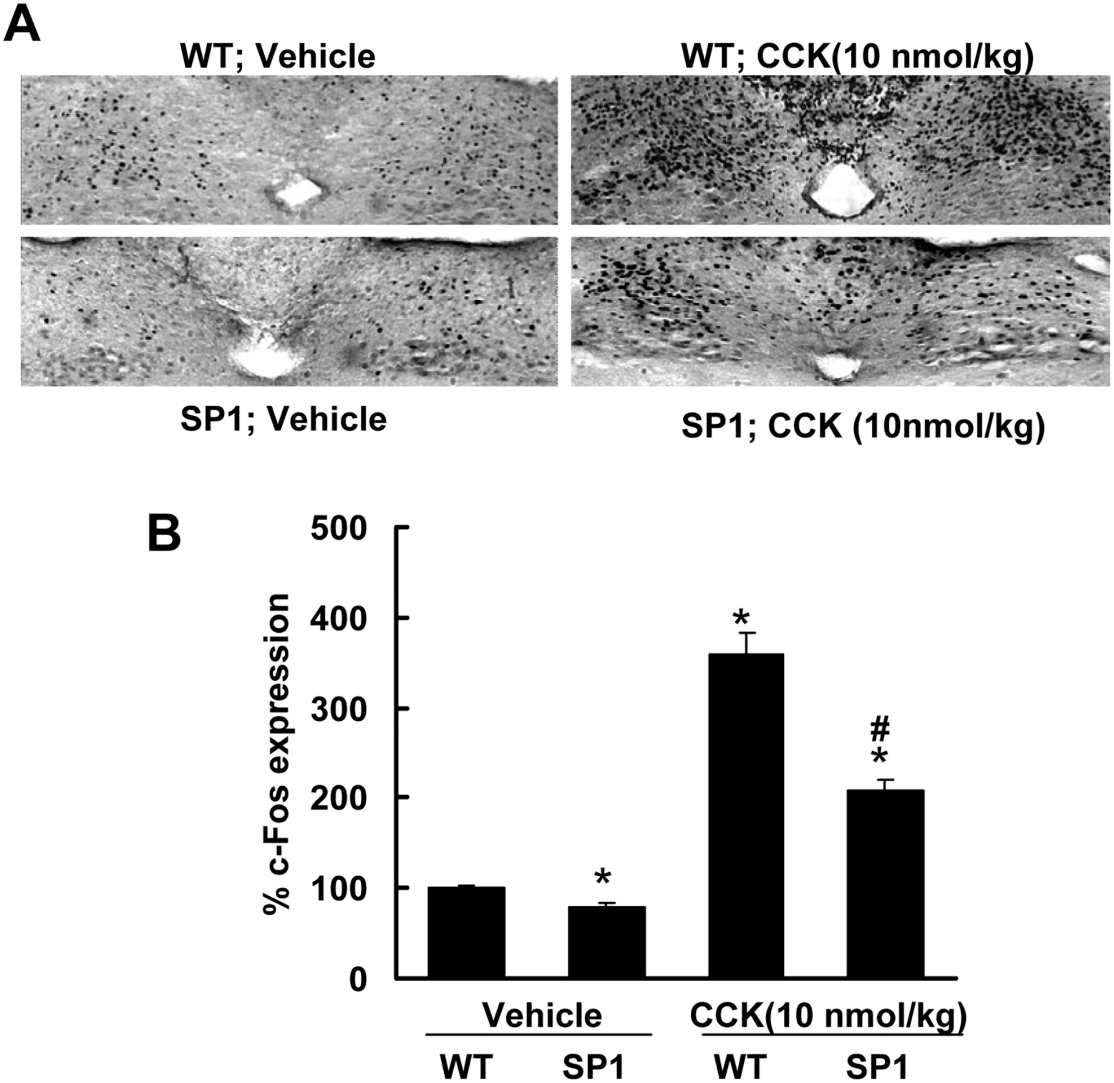
Synphilin-1 attenuated CCK-induced c-Fos activation in DVC in SP1 mice. WT control and SP1 mice at obese stage were subjected to c-Fos expression assays. A. Representative images of c-Fos immunostaining in NTS and AP areas. CCK increased c-Fos expression in DVC area in both WT and SP1 mice. B. Quantification of c-Fos positive immunostaining cells in NTS and AP areas. There was less c-Fos expression in DVC area in SP1 mice than in WT controls. * *P*<0.05 by two-way ANOVA, vs untreated WT mice. # *P*<0.05 by two way ANOVA, vs treated WT mice.

Previous studies have shown that doses of CCK that decrease feeding result in NTS neural activation [22,26]. NTS in the caudal brain stem plays a critical role in feeding behaviors related to the control of meal size [27]. To assess whether SP1 alters NTS neural activation in response to CCK, the patterns and levels of c-Fos activation in response to CCK were compared in control and obese SP1 mice. The baseline of c-Fos expression in SP1 mice is slightly lower than WT mice (Fig 5). CCK significantly increased c-Fos expression in the NTS and area postrema (AP) in both WT and SP1 mice. However, the increase in the WT controls was much greater than in the SP1 mice (Fig 5). SP1 reduced CCK-induced c-Fos activation in DVC area at both pre-obese and obese stages with a similar expression pattern compared with age matched WT controls.

## Discussion

The major finding of this study is that SP1 mice are deficient in their response to exogenous CCK both in terms of the effect on food intake and hindbrain neural activation. In contrast, SP1 overexpression did not alter feeding responses to amylin and GLP-1. Our previous studies demonstrate that hyperphagia in SP1 mice results in obesity since SP1 mice are pair-fed with the same amount of food as WT mice for 10 weeks, which can totally prevent the SP1-induced obesity [1]. Given that CCK reduced food intake by reducing meal size [14,15], our results showed that overexpression of SP1 in neurons caused a deficiency in response to CCK in brains, resulting in abolishing the CCK effects on food intake. These data suggest that synphilin-1-induced CCK response deficiency may contribute to the overall hyperphagia in SP1 mice.

Gastrointestinal peptides are released in response to nutrition intake. Meal termination and satiety are partly due to the negative feedback mediated by certain gastrointestinal peptides including amylin, GLP-1, and CCK [12–14,28]. Amylin plays an important role in satiety. Amylin can reduce food intake in mice under both nonfood-deprived and food-deprived conditions, both diabetic or non-diabetic status, as well as in both obese or lean mice [29,30]. Consistent with these findings, our results showed that amylin injection significantly reduced food intake in both pre-obese and obese SP1 mice in a dose-dependent manner. There was no difference in the response to amylin treatment between WT and SP1 mice. These results indicate that SP1 did not alter the amylin-linked satiety signaling.

GLP-1 is another gastrointestinal peptide secreted in response to food ingestion that negatively regulates food intake via activating the GLP-1 receptor [13,19]. Central or peripheral administration of long-acting GLP-1 agonists reduces food intake and leads to weight loss in diabetic rodents and humans [19,31]. Consistent with these findings, our results showed that exendin-4 (a GLP-1 R agonist) reduced food intake in both WT and SP1 mice. There was no difference in the feeding inhibitory response to exendin-4 between SP1 mice and age matched controls at both pre-obese and obese stages. This result suggests that there is no defect in GLP-1-linked satiety signaling in SP1 mice.

CCK is an anorectic gastrointestinal peptide released in response to food ingestion. CCK mediated satiety signals act through CCK1 receptors on vagal afferent nerves resulting in neuronal activation in the nucleus of the solitary tract (NTS) in the hindbrain that receives the input from vagal afferents [25]. Our results show that peripheral administration of CCK (1–10 nmol/kg) significantly reduced a liquid diet intake of the WT mice but did not have an effect in SP1 mice. Moreover, SP1 overexpression significantly attenuated CCK-induced c-Fos expression in NTS in SP1 mice compared with WT mice.

SP1 overexpression induced hyperphagia is the primary behavioral change in SP1 mice [1]. Hyperphagia can occur via disturbance of multiple systems, represented by an increase in meal size and/or meal number. Previous studies show that meal size is increased in SP1 transgenic mice [4]. Meal size control depends on interactions among multiple systems. However, the primary system involves gut feedback signaling arising from the presence of ingested nutrients in the gastrointestinal tract. In SP1 mice, a decrease in the efficacy of CCK to reduce meal size and c-Fos expression in hind brains at least partly contributed to SP1-induced hyperphagia and obesity. The CCK response deficiency could be the result of a potential decrease in vagal afferent activation by CCK or a decreased NTS response to vagal afferent input. The site of action for amylin is the area postrema [32,33] while long acting GLP-1 agonists have been proposed to have both vagal and non- vagal direct actions in brain [19, 34]. Thus, the intact feeding inhibitory responses to both amylin and exendin-4 could bypass both vagal and NTS circuitry leaving the issue unresolved.

In conclusion, this study demonstrates that SP1 alters CCK satiety signaling and suggests that this alteration may contribute to the increased food intake and deficit in meal size control in SP-1 mice.
